# Bottom-Up and Cognitive Top-Down Emotion Regulation: Experiential Emotion Regulation and Cognitive Reappraisal on Stress Relief and Follow-Up Sleep Physiology

**DOI:** 10.3390/ijerph19137621

**Published:** 2022-06-22

**Authors:** Yulin Wang, Elke Vlemincx, Iris Vantieghem, Monica Dhar, Debo Dong, Marie Vandekerckhove

**Affiliations:** 1 Faculty of Psychology and Educational Sciences, Vrije Universiteit Brussel, 1050 Brussels, Belgium; monica.dhar@uantwerpen.be; 2 Faculty of Psychological and Educational Sciences, University of Ghent, 9000 Ghent, Belgium; 3 Department of Health Sciences, Vrije Universiteit Amsterdam, 1081 Amsterdam, The Netherlands; e.vlemincx@vu.nl; 4 Faculty of Medicine and Psychopharmacology, Vrije Universiteit Brussel, 1050 Brussels, Belgium; iris.vantieghem@uzbrussel.be; 5 Collaborative Antwerp Psychiatric Research Institute (CAPRI), University of Antwerp, 2000 Antwerp, Belgium; 6 Faculty of Psychology, Southwest University, Chongqing 400715, China; debo.dong@gmail.com; 7 Faculty of Arts and Philosophy, University of Ghent, 9000 Ghent, Belgium

**Keywords:** experiential, reinterpret, stress, negative affect, rapid eye-movement sleep

## Abstract

Emotional stress throughout the day is known to affect objective sleep physiology and subjective sleep quality. In the interplay between emotions and sleep, emotion regulation plays a critical role in the recovery from stressful, emotional events and subsequent sleep. While the effects of top-down emotion regulation strategies such as cognitive reappraisal on sleep have been studied before, the impact of bottom-up emotion regulation strategies such as experiential emotion regulation is understudied. Cognitive reappraisal reflects the cognitive reinterpretation of the meaning of a stressful event, while experiential emotion regulation involves an active, non-intervening, accepting, open and welcoming approach of acknowledging awareness of raw sensory affective experiences or ‘experiential awareness’ in a first phase and expression in a second phase. The present study aims to investigate the effects of experiential emotion regulation and cognitive reappraisal on the recovery from pre-sleep emotional stress measured by (1) negative affect and (2) sleep structure. Sleep of forty-three healthy Dutch-speaking participants (22 females, 21 males) has been assessed using EEG polysomnography. Stress was triggered using a pre-sleep emotional failure induction, after which emotion regulation by experiential emotion regulation versus cognitive reappraisal versus control was induced twice. The control condition consisted of the reallocation of attention towards the neutral aspects of the emotional event. The results indicated that recovery from negative affect of the failure experience after single or repeated deployment of experiential emotion regulation and cognitive reappraisal was not significantly different from the control condition. Moreover, after repeated deployment, sleep physiology did not significantly differ between experiential emotion regulation, cognitive reappraisal, and the control condition in the impact of the regulation of the failure experience. The implications of the distinctive impact of experiential emotion regulation and cognitive reappraisal on both the pre-sleep emotional experience and follow-up sleep physiology are discussed.

## 1. Introduction

### 1.1. Emotion and Sleep

Sleep plays a crucial role in mental and physical health throughout one’s lifespan. On the one hand, sleep loss worsens mood and decreases the ability to regulate negative emotions [1,2]. It increases negative emotions and emotional reactivity, reduces the experience of positive emotions, and alters how individuals understand, express, and modify these emotions [3,4]. Sleep deprivation may even impede the effectiveness of adaptive emotion regulation, such as distraction and cognitive reappraisal, consequently impacting emotional well-being [5,6]. On the other hand, daily emotional stress is also known to affect subsequent sleep [7,8,9,10,11]. Emotional stress induced by an emotional failure experience does not only result in high negative affect, but can also affect sleep efficiency (SE), sleep onset latency (SOL), wake after sleep onset (WASO), total time awake, number of awakenings during the night, number of awakenings from rapid eye movement (REM) sleep (dream sleep characterized by REM, but also low muscle tone and rapid, low voltage waves), % REM-sleep, and % slow wave sleep (SWS) [10]. Although it has been consistently shown that emotional stress can elicit profound and lasting effects on sleep (for a review, see [12]), the moderating effects of emotion regulation remain relatively less understood.

### 1.2. The Moderating Effects of Emotion Regulation

Emotion regulation refers to the process by which an individual influences the nature of his or her emotions and how emotions are experienced and expressed [13,14]. Individuals may not always hold the ability to control the stress factors they encounter in life, but they may be capable of learning to adopt efficient emotion regulation strategies, and thus control the detrimental effects of stress on their sleep activity. Although often based on subjective reports, there is a growing interest in the potential role of emotion regulation in response to daily emotional events on sleep quality and vice versa (for examples, see [5,15]). However, prior research mostly focused on traditional top-down emotion regulation strategies, such as cognitive reappraisal and distraction, and less on bottom-up emotion regulation strategies, such as experiential emotion regulation. As a complementary bottom up emotion regulation strategy, experiential emotion regulation involves an active, non-intervening, accepting, open and welcoming compassionate approach towards the bodily felt sensory affective experience or ‘experiential awareness’ in a first phase, and its verbalization or ‘experiential expression’ in a second phase. To address this gap in the literature, our lab conducted a study to investigate whether experiential versus cognitive emotion regulation strategies moderate the relationship between emotions elicited by a painful failure experience and subsequent sleep differently [11]. Participants who were instructed to deploy experiential emotion regulation were asked to focus on their low-level and concrete affective experience by affectively acknowledging and understanding it in the context of the specific situation, and expressing it in an open, non-evaluative mode. Compared to participants deploying a cognitive emotion regulation strategy, those in the experiential emotion regulation condition took longer to fall asleep, but experienced significantly fewer awakenings, showed longer total sleep time (TST), and higher sleep efficiency (SE). The present study aimed to further validate the impact of bottom up experiential emotion regulation versus cognitive reappraisal on pre-sleep emotional experience and follow-up sleep physiology, both relative to a control condition.

#### 1.2.1. Top-Down Cognitive Reappraisal

A well-studied cognitive emotion regulation strategy is cognitive reappraisal. Cognitive reappraisal is defined as the attempt to reinterpret an emotion-eliciting situation in a way that alters its meaning and changes its emotional impact [16]. One approach to top-down cognitive reappraisal, ‘cognitive reinterpretation’, is a cognitive-linguistic strategy that alters the trajectory of emotional responses and diminishes its impact by changing the meaning of an emotion through reframing the context of the emotion-evoking stimulus [13] (e.g., “She did not say hello because she was distracted, not because she does not like me”). The theoretical models underlying cognitive emotion regulation, i.e., the process model of emotion regulation ([17]; for an alternative, see [18]), consider cognitive reappraisal as a linear top-down process. Consequently, most of the insights gained in the cerebral architecture of cognitive emotion regulation concern the downregulation by, and interplay between, prefrontal control circuits and limbic emotion generation circuits, such as the amygdala, striatum, and insula [19,20,21]. Given the adaptive effect of cognitive reappraisal on the modulation of emotional stress, we assume that cognitive reappraisal would also have a beneficial effect on sleep in the present study. However, research on the impact of cognitive reappraisal on sleep shows inconsistent findings. Some researchers found that poorer sleep quality is associated with a lower ability to cognitively reappraise [22]. Conversely, individuals who are more capable of adopting cognitive reappraisal in their daily lives are more likely to have enhanced sleep quality [23]. On the other hand, the impaired quality of sleep influences the use of emotion regulation, such as expressive suppression. According to Ellis, Prather, Grenen, and Ferrer (2020) [24], sleep quality was indirectly related to the habitual use of cognitive reappraisal. Moreover, other research demonstrated that sleep quality is not related to cognitive reappraisal ability [25,26]. Altogether, it is still unclear how cognitive reappraisal impacts pre-sleep emotional stress and thus contributes to follow-up sleep.

#### 1.2.2. Bottom-Up Experiential Emotion Regulation

Experiential emotion regulation involves an active, non-intervening, accepting, open and welcoming approach of acknowledging and gaining awareness of raw sensory affective experiences or ‘experiential awareness’ in a first phase and expression in a second phase. Instead of emphasizing an active and cognitively controlling way of coping with emotions, experiential emotion regulation highlights the importance of the affective process itself, as an adaptive signaling mechanism and a bottom-up pathway to process emotional experiences in more depth. Originating from humanistic, client-centered and experiential psychotherapeutic approaches, such as Experiential Focusing [27,28,29], experiential emotion regulation primarily focuses on the importance of experiencing one’s feelings and emotions in the immediate present to achieve emotional change. ‘Experiencing’ involves the awareness of an emotionally tinged experience together with its personal meaning [27]. Furthermore, experiential emotion regulation involves an accepting, non-intervening, open, and welcoming approach towards the present affective sensory feeling [28,29,30]. Research demonstrated that participants who were instructed to apply an ‘experiential self-focus’ on the concrete “what” or content of the feeling or affective experience were more adaptive to recover from painful life events compared to participants with a cognitive analytical “why” focus [31]. As illustrated by these findings, an experiential approach enhances people’s ability to face bottom-up generated stressors involving a bodily felt affective experience, leading to integration and acceptance of predictable stressors and reduced arousal levels from the stressors in the long-term.

### 1.3. Repeated and Sustained Emotion Regulation

In daily life, emotional stressful life events mostly involves repeated or persistent experience or confrontations with the same or similar emotional events (e.g., the memory of a deceased loved one, a divorce, or failing an important exam). Yet, in current emotion regulation research, only a few studies investigated the effects of repeated or sustained emotion regulation, which was also limited to cognitive strategies. For instance, Erk et al. (2010) [32] investigated the neural activity associated with the temporal dynamics of acute and sustained cognitive emotion regulation in patients with major depressive disorder and healthy controls using functional MRI (fMRI). Only amongst healthy controls, sustained cognitive emotion regulation was associated with a reduced activation in the amygdala after a 15 min delay. In a study by Denny et al. (2015) [33], cognitive reappraisal resulted in decreased negative emotion and amygdala activation, which remained attenuated for emotional images that had been reappraised four times, compared to images that were reappraised once, new control images, and control images that were never reappraised. Another study showed that repeated cognitive reappraisal resulted in reduced negative feelings and stronger dorsolateral and ventrolateral prefrontal cortex responses one day later [34]. Importantly, acute versus repeated emotion regulation effects may be different for cognitive and experiential emotion regulation. When applying a cognitive approach to emotional stressors, the emotion itself might be immediately regulated [11]. Conversely, experiential emotion regulation may initially enhance affective intensity and reactivity when processing a negative emotional stressor, whereby only repeated processing of the same or similar emotional stressors may result in profound regulation and recovery [12,35]. This adaptive regulation and recovery processes are expected to result in less emotional intensity and less negative evaluations of similar stressful events in the future.

### 1.4. The Present Study

Current emotion regulation research is lacking an understanding of (1) the different moderating effects of cognitive-versus experiential emotion regulation of emotional challenges and (2) the differential effects of single-versus repeated emotion regulation. To address these goals, our lab conducted a study to investigate whether experiential versus cognitive emotion regulation strategies differentially moderate the relationship between emotions elicited by a painful failure experience and subsequent sleep [11]. Participants who deployed experiential emotion regulation, compared to participants deploying a cognitive emotion regulation strategy, took a longer time to fall asleep, but experienced significantly fewer awakenings, showing a longer total sleep time (TST) and higher sleep efficiency on the other hand. The present study aimed to (1) replicate these effects, (2) and investigated the impact of single versus repeated experiential emotion regulation and cognitive reappraisal on emotional experience and sleep physiology. The study also focused on sleep physiology outcomes specific to REM-sleep, such as the number of rapid eye movements, REM sleep fragmentation assessed by the arousal index, and the number of awakenings during REM sleep [36,37], as REM sleep particularly contributes to the regulation of emotional distress.

We predicted that, in response to a bottom-up generated emotion (i.e., a failure experience), cognitive reappraisal elicits an immediate emotion regulation impact, but is not more effective after repeated regulation, whereas experiential emotion regulation is more effective after repeated regulation. The following hypotheses were formulated on the differential impact of repeated deployment of cognitive versus experiential emotion regulation on negative affect in response to a negative emotional failure experience: (1) after a first time of emotion regulation, we expected cognitive reappraisal to reduce negative affect more than experiential emotion regulation and the control-condition, whereas (2) after a second, repeated, time of emotion regulation, we expected that experiential emotion regulation to reduce negative affect more than cognitive reappraisal. In addition, to test the following a priori hypothesis about the differential impacts of cognitive versus experiential emotion regulation on sleep physiology was formulated: after repeated emotion regulation, we expect that experiential emotion regulation result in less fragmented (e.g., higher sleep efficiency, higher total sleep time, lower wake percentage) and less arousing sleep physiology (e.g., less ‘restless REM sleep’) than cognitive reappraisal and a control condition. In line with our previous study [11], the moderating effects of gender and order of the nights (failure night on the second night and baseline night on the third night versus the reversed order) were also explored in the analyses to test the above hypotheses.

## 2. Materials and Methods

### 2.1. Participants

The sample size was determined according to a previous study (N = 28 for two groups) conducted in the lab directly compared the effects of experiential to cognitive emotion regulation strategies on pre-sleep stress and subsequent sleep [11]. Forty-four participants were recruited via various sources, such as mailing lists of the electronic communication platform of the Vrije Universiteit Brussel (VUB), advertisements on the Etterbeek campus of the VUB, and the VUB participation pool. Participants were selected and screened by means of the Mini International Neuropsychiatric (semi-structured) Interview (M.I.N.I.) [38] to exclude participants with psychiatric and psychological problems. Moderate to good sleepers were selected based on the Pittsburgh Sleep Quality Index (PSQI), using a cut-off score of 5 [39]. Furthermore, before the study, participants were asked to keep a two-week sleep diary to check for irregular sleep–wake schedules and were asked to maintain their normal sleep–wake patterns for two weeks before the experiment. Habitual short or long sleepers were excluded based on their sleep diary. Other exclusion criteria included pregnancy, shift work, medication intake known to influence sleep, a BMI higher than 28, and consuming three or more alcoholic or caffeinated beverages a day. One participant was excluded due to symptoms of periodic limb movement disorder. Finally, 43 healthy Dutch-speaking participants, ranging from 18 to 36 years in age (mean age = 24.28 years, SD = 4.99), participated in this study. These participants were randomly assigned (using the MATLAB function “randperm (3)” to generate a random serial for every three participants) to one of three experimental groups: experiential emotion regulation (n = 15, 8 males, 25.00 ± 5.93 years), cognitive reappraisal (n = 13, 5 males, 25.77 ± 5.22 years), and control (n = 15, 8 males, 22.27 ± 3.04 years). Age (F (2, 40) = 2.06, *p* = 0.14, also see Table 1) and gender (X^2^ (2, N = 43) = 0.81, *p* = 0.67) did not differ among the three experimental groups. Each participant received EUR 120 for their participation. Informed consent was obtained from all participants. The Ethics Committee approved the study at the VUB, and all procedures involved were consistent with the sixth revision of the Declaration of Helsinki. 

### 2.2. Self-Report Measures

The Emotional Approach Coping Scale (EACS; [40]) was utilized to assess individual differences in emotional processing and emotional expression. The EACS contains 16 items, and each item was answered by the participants on a 4-point Likert scale ranging from 1 (never) to 4 (always). The Emotion Regulation Questionnaire (ERQ; [16], Dutch version) was used to assess individual differences in cognitive reappraisal and suppression. The ERQ consists of 10 items, and each item was answered by the participants on a 7-point Likert scale ranging from 1 (strongly disagree) to 4 (strongly agree). The Toronto Alexithymia Scale (TAS-20; [41]) was used to measure individual differences in alexithymia by (1) the capacity to identify feelings and to distinguish them from physiological sensations, (2) the capacity to communicate those feelings to others, and (3) the tendency to exhibit externally oriented thinking. The TAS consists of 20 items, giving participants 5 choices for each item from 1 (strongly disagree) to 5 (strongly agree). The Pittsburgh Sleep Quality Index (PSQI; [39]) was used to assess self-reported sleep quality and disturbances over the course of 1 month. The PSQI includes 19 items that generate seven component scores: subjective sleep quality, sleep latency, sleep duration, habitual sleep efficiency, sleep disturbances, use of sleep medication, and daytime dysfunction. Finally, the Positive and Negative Affect Schedule (PANAS; [42]) was used to measure state changes for both positive and negative affect. The PANAS has 20 items, giving participants 5 choices for each item from 1 (very slightly or not at all) to 5 (extremely). Participants completed all of these questionnaires via LimeSurvey (https://www.limesurvey.org/). All of the above-mentioned questionnaires have high internal consistency and test–retest reliability, as well as convergent and discriminant validity.

### 2.3. Procedure

Two weeks prior to the start of the study, participants were asked to maintain their habitual sleep–wake patterns. After these two weeks, participants spent three nights of 8 h in bed in the sleep laboratory of the VUB. The first night, the adaptation night, included a diagnostic sleep study to rule out any sleep disorders and allow the participants to adapt to the laboratory procedures and environment. This was followed by a baseline night and an experimental night. The experimental night involved a ‘failure’ induction, after which 15 participants were trained in using experiential emotion regulation, 13 participants were trained in using cognitive emotion regulation, and 15 participants were instructed to use a non-specific regulation to process their emotional stress as a control condition. The order of the baseline and experimental night was counterbalanced.

**Adaptation and baseline nights.** On the days of the adaptation and baseline nights, participants arrived at the lab at 8:00 p.m. After a more detailed explanation of the goal of the study as well as the procedure, the participants completed the informed consent forms. On both the adaptation and the baseline night, EEG electrodes were attached, after which the PANAS was administered. Between 9:00 p.m. and 11:00 p.m., only recreational activities (e.g., reading relaxing literature) were allowed. At 10:45 p.m., a wake-EEG was recorded. At 11:00 p.m., the participants were asked to go to bed. At 7:00 a.m. the next morning, participants were awakened and completed the PANAS. At 7:15 a.m., electrodes were removed, and breakfast was served. Finally, at about 8:00 a.m., the participants left the lab.

**Figure 1:**
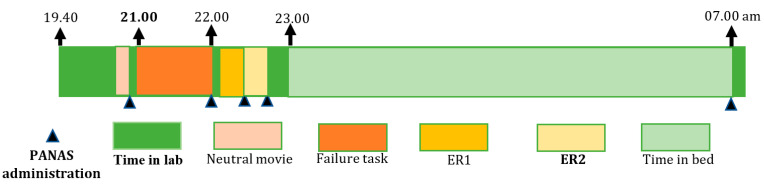
The timeline set-up for the experimental failure night. Abbreviations: PANAS = Positive and Negative Affect Schedule; ER1 = the first time of emotion regulation; ER2 = the second time of emotion regulation.

**Experimental night.** On the day of the experimental failure night (see Figure 1 for the timeline set-up), participants arrived at the lab at 8:00 p.m. where EEG electrodes were attached, after which the PANAS was administered. At 9:00 p.m., an awake-EEG was recorded. The failure induction consisted of failure feedback in response to different cognitive tasks, which we introduced as an intelligence test [43,44]. In several previous pilot tests, we adapted the level of difficulty so that all participants had a minimum score of between 0 and 3, whereas the maximum score of the test was 9 to 11. We validated the test in the first 12 participants. The tasks and failure feedback were presented on a computer to standardize the presentation and individualize the scores based on their real performance. After attaching the electrodes, participants were asked to watch a five-minute emotionally neutral film clip (about nature), which served as a baseline [45]. After participants rated their emotional state with the PANAS again, the experimenter entered the room to introduce the procedure of the “intelligence test” that the participants had to perform. The experimenter informed the participants that they would be participating in a new cognitive test reflecting their level of intelligence and predicting future professional achievement. They were told: “It is very important that you concentrate and do this intelligence test to the best of your ability. The test is being developed for international use and reflects your potential regarding general intelligence and professional achievements.” The test contained five subtests: (1) spatial ability tasks, (2) logical steps tasks (e.g., figure completion and mathematical problem-solving tasks), (3) a numeric and visual memory test, (4) a semantic reasoning task, and (5) an impossible semantic test. The impossible semantic test consisted of the presentation of three unrelated words ten times, whereby the participants had to find the related word (e.g., blood, music, cheese). Between the different tasks, the experimenter entered the room several times, giving comments with an annoyed and irritated tone of voice. The first time, the following feedback was given: “You are moving around too much, causing physiological artifacts and the data to become useless. Please sit still.” After five minutes, the experimenter entered the room again, sighing and appearing annoyed, checking the electrodes, and then leaving the room without talking to the participant. After each subtest and at the final competition of the test, the participant’s scores were presented on a computer screen accompanied by failure feedback stating that the person’s test achievements were weak and below average. Upon completion of the test, the experimenter informed the participants that the physiological measurements were useless so far. However, in reality, no physiological data were obtained, and the test was far too difficult with too little time available to do well.

**Emotional experience.** After the emotional failure induced by the “intelligence test”, emotion regulation was completed twice by a writing task. Depending on the experimental group, the participants received the following instructions:

We can see in your physiological data that you are very stressed. Several artifacts are visible. Because we want you to have a good night, we would like you to apply (1) experiential emotion regulation: “write about your emotional experience, the feeling you get from the tasks, and your related performance. Focus on your bodily felt affective feelings, allowing it to come into your awareness, welcome and accept this experience and stay with it even if it feels negative”,(2) cognitive reappraisal: “reinterpret the test, your performance and feedback you received and give it another meaning in such a way that you feel better”; or in the (3)—control condition: “write about the tasks you just completed. Describe them. What were the characteristics of these tasks? You have to focus only on the practical aspects of the tasks.” To check whether the participants actually used the specific induced emotion regulation strategies successfully, the experimenter carefully assessed what the participants had written down. All participants understood the writing instructions well and reflected on their failure experience according to the given instructions.

Immediately after the first emotion regulation moment (“emotion regulation moment 1”), participants were asked to fill in the PANAS again. Just before sleep time, participants were asked to repeat the same exercise a second time (“emotion regulation moment 2”), whereafter they completed the PANAS again. After their morning awakening at 7:00 a.m., the PANAS was administered once more. Next, the electrodes were removed, and before leaving the lab, a “funneled debriefing” procedure was adopted. It was emphasized that the tasks were, in reality, far too difficult and that this made their performance unrelated to intelligence and academic or career success. They were told: “The task you performed yesterday is, in general, far too difficult to fulfill in the given time. Everyone obtains a score between 0 and 3, which means that your score was not bad at all and that we definitively have to adapt our tests to a more normal level of difficulty.” It was explained that the task was not a task of intelligence, but part of a negative emotion induction procedure. Moreover, participants were informed that the experimenter was intentionally irritated to enhance their failure experience to facilitate the overall goal of the study, which was to test the effectiveness of emotion regulation strategies by the writing procedure during the recovery from an emotional, negative experience. The debriefing was conducted in a way that participants really understood the true nature of the study and the manipulations, and they felt good again. After the debriefing, breakfast was served. At about 8:00 a.m., participants left the lab.

### 2.4. Polysomnography

The polysomnography setup consisted of six EEG electrodes (F3, C3, O1, F4, C4, O2) referenced to a single mastoid, two electro-oculogram (EOG) electrodes referenced to a single mastoid (LOC-ROC), a bipolar submentalis electromyogram (EMG), a tibialis EMG, and electro-cardiogram (ECG) electrodes. Belts were located on the thorax and abdomen to record the participant’s breathing rhythm during the experiment. A 32 channel Embla N7000 recording system was used (Medcare) with a DC offset of 500 mV max, and a fixed DC low cut filter at 0.3 Hz. The signal was digitized at a sampling rate of 500 Hz using Somnologica 3 system (version II Software, Medcare Flaga, Reykjavik, Iceland). EEG and EOG signals were high pass filtered at 0.5 Hz and low pass filtered at 40 Hz; EMG channels were high pass filtered at 5 Hz and low pass filtered at 70 Hz. Sleep recordings were scored in 30 s epochs according to the American Academy of Sleep Medicine (AASM, 2013) rules into the REM (stage R), wakefulness (stage W), and NREM stages 1 (N1), 2 (N2), 3 (N3). The data were scored blindly by two trained specialists reaching an inter reliability of 90%. Sleep stages were scored based on the new guidelines of the AASM (2013) with the following sleep variables: total sleep time (TST), sleep onset latency (SOL: from the moment the lights are out until the first epoch of any sleep), total wake time (% wake: the amount of wake time during the total recording time in minutes after sleep onset), time awake after sleep onset (WASO), sleep efficiency (SE: the total sleep time divided by the time in bed [TST/TIB]), % of slow wave sleep (SWS: defined as stage N3), % REM sleep (defined as stage R), REM latency (defined as sleep onset to first epoch of REM sleep), % stage N1, and % stage N2. All epochs containing movements or EMG artifacts were excluded from the analysis. Beyond these parameters, we additionally calculated the number of awakenings during REM sleep, the arousal index (ArI; the average number of arousals per hour, regardless of the source of those arousals was scored based on the criteria set by American Sleep Disorders Association Atlas Task Force [46]), and REM density (defined as the number of eye movements for each 30 s epoch of REM sleep). Differences in REM-related sleep indices illustrate the difference between both emotion regulation conditions in how they reduce emotional stress during REM-sleep [37,47].

## 3. Data Analysis and Results

### 3.1. Detailed Report of the Dataset

The dataset of this study contained missing data points. One participant did not fill in the ERQ and the EACS questionnaires, and four participants did not complete the TAS-20 questionnaire. Due to technical problems, the arousal index of four participants (one participant in the experiential emotion regulation group, one participant in the control group, and two participants in the cognitive reappraisal group) could not be calculated, both for the baseline and experimental night. Due to these recording problems, the number of rapid eye movements of 12 participants during the baseline night could not be calculated (five participants in the experiential emotion regulation group, three participants in the neutral control group, and four participants in the cognitive reappraisal group). In addition, the number of rapid eye movements of 19 participants during the experimental night (six participants in the experiential emotion regulation group, five participants in the control group, and eight participants in the cognitive reappraisal group) could not be calculated. As there were too many missing data points in the number of rapid eye movements during REM sleep for both the baseline and experimental night (also see Table 1 and Table 2), we excluded this index from further data analysis.

Afterwards, distributions were examined parameter by parameter. Several outliers on some sleep parameters were detected using absolute deviation from the median [48]. Firstly, the total sleep time of two participants (in the cognitive reappraisal group with values of 207 min and 2 min) on the baseline night, and the total sleep time for another two participants (in the cognitive reappraisal group with values of 277 min and 204 min) on the experimental night, were left out based on the exclusion criteria of outliers (a deviation of three units). Secondly, the sleep onset latency (167 min), wake after sleep onset time (106 min), wake percentage (34%), and sleep efficiency (66%) for one participant in the cognitive reappraisal group during the baseline night were left out based on the exclusion criteria of outliers (a deviation of three units). Thirdly, the wake percentage (25%) and sleep efficiency (75%) for one participant in the control group during the experimental night were left out based on the exclusion criteria of outliers (a deviation of three units).

### 3.2. Comparison of the Three Experimental Groups at Baseline

To determine the effects of different emotion regulation strategies on both negative affect and follow-up sleep physiology, we performed a one-way between-subjects ANOVA to check if there were significant differences between the three experimental groups in (1) self-reported sleep quality, coping, and emotion regulation strategies; (2) sleep physiology during the baseline night; and (3) negative affect measured at the baseline night and after the baseline movie during the experimental night. The results indicated that there were no significant differences between the three groups in sleep physiology on the baseline night (see the detailed reports of the statistics in Table 1), nor in self-reported sleep quality (F (2,40) = 0.02, *p* = 0.99, also see Table 1). Moreover, no significant differences were found between the three experimental groups for coping and emotion regulation ability, including cognitive reappraisal, emotional suppression, emotional processing, emotional expression, difficulty describing and identifying feelings, and externally oriented thinking (also see Table 1 for more details). Finally, the three groups did not significantly differ with regard to negative affect on the baseline night (F (2,40) = 0.44, *p* = 0.65, also see Table 1), nor regarding negative affect after watching the baseline movie on the experimental night (F (2, 18.70) = 1.41, *p* = 0.27, also see the descriptive statistics in Table 2).

### 3.3. Manipulation Check

A repeated measures ANOVA was conducted in JASP (https://jasp-stats.org/, accessed on 20 March 2020) to test whether the failure experience elicited negative affect in all three groups, and negative affect decreased after the failure experience with emotion regulation in all three groups. We entered negative affect (NA) as the dependent variable at different moments. The within-subjects independent variable, defined as ‘time’, has four levels: after the baseline movie, after the failure task, after the first time of emotion regulation (ER1), and after the second time of emotion regulation (ER2) before sleep. Mauchly’s test of sphericity indicated that the assumption of sphericity was violated, indicating that there was a significant difference in the variances of the within-subject factor. As such, we completed sphericity corrections with Greenhouse–Geisser correction. All *p*-values of the post hoc analysis comparing negative affect during the four-time moments with a significant main effect of time were Bonferroni corrected.

**Figure 2:**
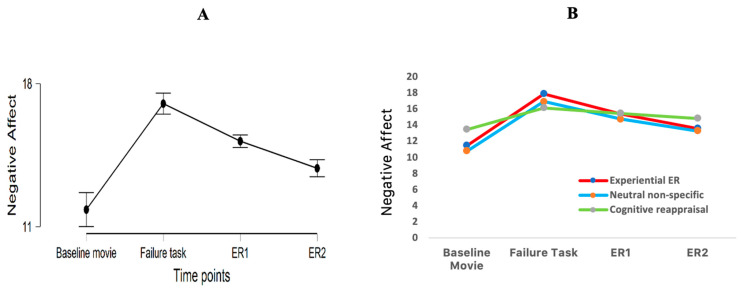
(**A**). Negative affect (NA) reported at different time moments: NA after the baseline movie; NA after the failure task; NA after the first time of emotion regulation (ER1) and NA after the second time of emotion regulation (ER2). (**B**). NA reported at different time moments for each experimental group.

A significant main effect of time was found (F (1.57, 65.90) = 15.60, *p* < 0.001, η^2^_p_ = 0.40) (see Figure 2). Post hoc analysis revealed that negative affect after the failure task was significantly higher compared to negative affect after the baseline movie (t (42) = 4.82, *p* < 0.001, Cohen’s d = 0.0.74), and after ER1 (t (42) = 5.06, *p* < 0.001, Cohen’s d = 0.77). Negative affect after ER2 was significantly lower than after ER 1 (t (42) = 3.80, *p* = 0.003, Cohen’s d = 0.58). There were no significant differences between negative affect after the baseline movie and negative affect after ER2 (t (42) = 2.14, *p* = 0.23, Cohen’s d = 0.33). Therefore, the failure experience elicited negative affect in all three experimental groups, and negative affect reduced across time after the induction.

### 3.4. Effects of Emotion Regulation on Negative Affect

As our hypotheses predict a significant difference between emotion regulation strategies depending on the moment the emotion regulation has taken place, we conducted an ANOVA with negative affect as dependent variable and four independent variabless: emotion regulation condition (cognitive reappraisal versus experiential emotion regulation versus control, between-subjects), time (baseline versus stressor versus ER1 versus ER2, within-subjects), gender (male versus female, between-subjects) and order of nights (baseline night on the second night and failure night on the third night versus the reversed order, between-subjects). All ANOVA results were Greenhouse–Geisser corrected where appropriate. The results showed that the effect of time was statistically significant, F (1.59, 50.96) = 8.64, *p* = 0.001, η^2^_p_ = 0.21. However, the interaction between time and emotion regulation condition was not significant. The interaction between time and gender, the interaction between time and order, the three-way time x emotion regulation condition x gender interaction, the three-way time x emotion regulation condition x order interaction, and the time x emotion regulation condition x gender x order interaction were also not significant (all Ps > 0.05).

Since no significant interaction between emotion regulation condition and time was found, our hypothesis that experiential emotion regulation differs from cognitive reappraisal depending on the emotion regulation moment could not be supported. To generate new hypotheses for future studies, specific exploratory post hoc analyses were performed, exploring the predicted effects of emotion regulation condition on decreases in negative affect after both emotion regulation moments.

#### 3.4.1. Immediate Effects of Emotion Regulation on Negative Affect

To examine the immediate effects of emotion regulation on the recovery from the failure experience, the decrease in negative affect from the failure experience to the first time of emotion regulation was entered as dependent variable (also see Table 2) in a one-way between-subjects ANOVA with emotion regulation condition (cognitive reappraisal, experiential emotion regulation, control) as independent variable. There was no statistically significant effect of emotion regulation condition, F (2, 40) = 2.35, *p* = 0.11, η^2^_p_ = 0.11 (also see Figure 2B). Therefore, the hypothesis that after a first emotion regulation moment, cognitive reappraisal would reduce negative affect more strongly than experiential emotion regulation and a control condition could not be confirmed.

#### 3.4.2. Repeated Effects of Emotion Regulation on Negative Affect

To examine the repeated effects of emotion regulation on the recovery from the failure experience, the decrease in negative affect from the failure experience to the second time of emotion regulation was entered as dependent variable (also see Table 2) in a one-way between-subjects ANOVA with emotion regulation condition (cognitive reappraisal, experiential emotion regulation, control) as independent variable. Similarly, there was no statistically significant effect of emotion regulation condition, F (2, 40) = 1.91, *p* = 0.16, η^2^_p_ = 0.09 (also see Figure 2B). Therefore, another hypothesis—after a second, repeated, emotion regulation moment, experiential emotion regulation should reduce negative affect more than cognitive reappraisal and the control condition—could not be confirmed either.

### 3.5. Effects of Emotion Regulation on Sleep Physiology

To test whether the three experimental conditions had a differential impact on sleep physiology following a failure induction, analyses of covariance were conducted with multivariate analyses (MANCOVAs) to adjust for multiple testing and reduce the likelihood of Type I error. For the MANCOVAs, Pillai’s trace statistics were reported for violations of variance–covariance homogeneity [49]. Notably, as indicated in the correlational matrix of different sleep variables (see Supplementary Table S1), sleep efficiency (SE) is highly correlated with both % wake (r = −0.97) and WASO (r = −0.93), and WASO is highly correlated with % wake (r = 0.93). Therefore, WASO and % wake were not included in the multivariate analysis, given that sleep efficiency involves time awake after sleep onset and total wake time. The remaining 10 sleep dependent variables were grouped into three clusters to avoid over-fitting of the MANCOVA model due to the sample size (Babyak, 2004). Cluster 1 consisted of three sleep continuity variables (SOL, SE, TST), Cluster 2 consisted of five sleep architecture variables (%N1, %N2, %SWS, %REM, latency to REM), and Cluster 3 consisted of two arousing sleep physiology variables (arousal index, number of awakenings during REM sleep).

#### 3.5.1. Sleep Continuity

MANCOVA was used to compare the three experimental conditions on sleep continuity. The three sleep continuity variables were simultaneously entered as dependent variables. Experimental condition (cognitive reappraisal, experiential emotion regulation, control), gender, and order of nights were added as between-subjects variables, and the corresponding sleep variables of the baseline night were entered as covariates. No significant multivariate effect of condition on the combined three outcomes was found (F (6, 50) = 0.39, *p* = 0.88, η^2^_p_ = 0.04), suggesting that sleep onset latency, sleep efficiency, and total sleep time did not vary amongst experimental conditions.

#### 3.5.2. Sleep Architecture

MANCOVA was used to compare the three experimental conditions on sleep architecture. The five sleep architecture variables were simultaneously entered as dependent variables. Experimental condition (cognitive reappraisal, experiential emotion regulation, control), gender, and order of nights were added as between-subjects variables, and the corresponding sleep variables of the baseline night were entered as covariates. No multivariate effect of condition on the combined five outcomes was found (F (10, 48) = 0.73, *p* = 0.69, η^2^_p_ = 0.13), illustrating that there was no evidence that sleep architecture, including sleep stage N1, N2, N3, REM sleep, and latency to REM differed between experimental conditions.

#### 3.5.3. Arousing Sleep Physiology

MANCOVA was used to compare the three experimental conditions on arousing sleep physiology. Arousal index and number of awakenings during REM sleep were simultaneously entered as dependent variables. Experimental condition (cognitive reappraisal, experiential emotion regulation, control), gender, and order of nights were added as between-subjects variables, and the corresponding sleep variables of the baseline night were entered as covariates. No multivariate effect of condition on the combined two outcomes was found (F (4, 46) = 1.25, *p* = 0.31, η^2^_p_ = 0.12), showing no evidence for the notion that the arousal index and number of awakenings during REM sleep differed between experimental conditions.

#### 3.5.4. Post Hoc Exploratory Analysis of a Priori Formulated Hypotheses

The results did not demonstrate a significant effect of emotion regulation condition. To generate hypotheses for future studies, and to directly compare results to previous findings for replication purposes [11], we opted to further explore the effect of experimental condition for each sleep variable separately, using exploratory post hoc analyses. An ANCOVA was conducted for each sleep variable with experimental condition (cognitive reappraisal, experiential emotion regulation, control), gender, and order of nights as between-subjects variables, while the corresponding sleep variables of the baseline night were entered as covariates. The assumption of equal variance for the ANCOVAs was checked using Levene’s test. The results are reported using *p*-values and effect sizes quantified by partial eta square (η^2^_p_). In case of a significant main effect of the experimental condition with FDR correction for multiple comparisons, contrasts were set to compare experiential emotion regulation versus cognitive reappraisal, and experiential emotion regulation versus control condition. All other follow-up contrasts (e.g., cognitive reappraisal versus control condition) were corrected for multiple comparisons using Tukey’s test.

**Total sleep time.** Total sleep time measured on the baseline night did not act as a modulator: F (1, 27) = 2.16, *p* = 0.15, η^2^_p_ = 0.07. No significant main effect was found for emotion regulation condition (F (2, 27) = 0.35, pFDR = 0.76, η^2^_p_ = 0.03) and order of nights (F (1, 27) = 0.97, *p* = 0.33, η^2^_p_ = 0.04). However, a significant effect of gender was found (F (1, 27) = 4.83, *p* = 0.04, η^2^_p_ = 0.15). The interaction effect between the experimental condition and gender was found to be significant too: F (2, 27) = 5.50, *p* = 0.01, η^2^_p_ = 0.29. Women showed a longer total sleep time than men in the cognitive reappraisal condition: (F (1,7) = 12.27, *p* = 0.01, η^2^_p_ = 0.64, M_women_ = 455.90, M_men_ = 404.80), with no gender differences in the experiential emotion regulation condition (F (1,11) = 0.25, *p* = 0.63, η^2^_p_ = 0.02, M_women_ = 438.40, M_men_ = 440.80) or the control condition (F (1,12) = 0.0004, *p* = 0.98, η^2^_p_ = 0.00, M_women_ = 433.20, M_men_ = 433.90).

**Sleep efficiency.** Sleep efficiency measured on the baseline night did not have a modulating impact: F (1, 29) = 0.83, *p* = 0.37, η^2^_p_ = 0.03. Yet, a significant main effect of gender was found, F (1, 29) = 9.10, *p* = 0.005, η^2^_p_ = 0.24, but no significant main effects were found for the experimental condition (F (2, 29) = 5.27, pFDR = 0.06, η _p_^2^ = 0.27) or order of nights (F (1, 29) = 0.23, *p* = 0.64, η^2^_p_ = 0.008). The exploratory analysis indicated that women did not differ from men in sleep efficiency: t (40) = 1.86, *p* = 0.07, Cohen’s d = 0.54. Furthermore, the interaction effect between experimental condition and gender was found to be significant: F (2, 29) = 10.01, *p* < 0.001, η^2^_p_ = 0.41. Men showed a lower sleep efficiency than women in the cognitive reappraisal condition (F (1,9) = 23.64, *p* < 0.001, η^2^_p_ = 0.72, M_women_ = 97.97, M_men_ = 89.84). Neither the control condition (F (1,11) = 4.38, *p* = 0.06, η^2^_p_ = 0.29, M_women_ = 94.37, M_men_ = 97.44) nor the experiential emotion regulation condition (F (1,12) = 0.006, *p* = 0.94, η^2^_p_ = 0.00, M_women_ = 95.84, M_men_ = 96.21) found gender differences.

**Total wake time.** The percentage of time awake measured on the baseline night did not appear to be a modulator: F (1, 29) = 0.95, *p* = 0.34, η^2^_p_ = 0.03. A significant main effect of gender was found, F (1, 29) = 9.77, *p* = 0.004, η^2^_p_ = 0.25, but no significant main effects were found for experimental condition (F (2, 29) = 5.06, pFDR = 0.06, η_p_^2^ = 0.30) and order of nights (F (1, 29) = 0.13, *p* = 0.72, η^2^_p_ = 0.004). In addition, the exploratory analysis indicated that women did not differ from men in total wake time: t (40) = −1.95, *p* = 0.06, Cohen’s d = −0.57. In addition, the interaction effect between the experimental condition and gender was found to be significant: F (2, 29) = 9.77, *p* = 0.001, η^2^_p_ = 0.40. Women showed a smaller percentage of time awake than men in the cognitive reappraisal condition: (F (1,9) = 23.04, *p* < 0.001, η^2^_p_ = 0.72, M_women_ = 2.03, M_men_ = 10.16). There were no gender differences in the experiential emotion regulation condition (F (1,12) = 0.00026, *p* = 0.996, η^2^_p_ = 0.00) and the control condition (F (1,11) = 3.94, *p* = 0.07, η^2^_p_ = 0.26, M_women_ = 5.63, M_men_ = 2.70).

**Awakenings during REM sleep.** The number of awakenings during REM sleep during the baseline night was not significant as a modulator: F (1, 31) = 3.03, *p* = 0.09, η^2^_p_ = 0.09. A significant main effect of gender was found: F (1, 31) = 5.53, *p* = 0.03, η^2^_p_ = 0.15. However, no significant main effects were found for the experimental condition (F (2, 31) = 4.15, pFDR = 0.12, η^2^_p_ = 0.21) and order of nights (F (1, 31) = 1.94, *p* = 0.17, η^2^_p_ = 0.06). An exploratory analysis indicated that there was no gender difference in awakenings during REM sleep: t (41) = −1.95, *p* = 0.06, Cohen’s d = −0.57. In addition, the interaction effect between experimental condition and gender was significant, F (2, 31) = 4.30, *p* = 0.02, η^2^_p_ = 0.22. Men showed a higher number of awakenings during REM sleep compared to women (F (1,10) = 5.80, *p* = 0.04, η^2^_p_ = 0.37, M_women_ = 3.63, M_men_ = 10.80) in the cognitive reappraisal condition, while no gender difference was found in the experiential emotion regulation condition (F (1,12) = 0.08, *p* = 0.78, η^2^_p_ = 0.007, M_women_ = 5.43, M_men_ = 4.63), nor in the control condition (F (1,12) = 0.10, *p* = 0.76, η^2^_p_ = 0.008, M_women_ = 5.29, M_men_ = 4.63).

**Arousal index.** The arousal index measured at the baseline night appeared to be a significant modulator, F (1, 27) = 27.78, *p* < 0.001, η^2^_p_ = 0.51. The correlation between the arousal index at the baseline night and the arousal index at the experimental night is 0.74 (*p* < 0.001). The main effect of experimental condition was found to be not significant, F (2, 27) = 3.31, pFDR = 0.15, η^2^_p_ = 0.20. Neither the main effect of gender (F (1, 27) = 2.31, *p* = 0.14 η^2^_p_ = 0.08), nor that of order of nights (F (1, 27) = 0.07, *p* = 0.79, η^2^_p_ = 0.003) were significant.

No significant results were obtained for the emotion regulation condition, gender, or order of nights for the following sleep variables: SOL, WASO, %N1, %N2, %SWS (N3), %REM sleep, and latency to REM sleep (also see Table 2).

## 4. Discussion

Stress during the day impacts sleep physiology [7,8,9,50]. Although recent research begun to break down the complex interplay between emotion, emotion regulation, and sleep, the moderating impact of emotion regulation involved in the effects of emotion on sleep is still not well understood [51]. The current study aimed to study the modulating and differential impact of a more bottom-up approach, namely experiential emotion regulation, versus a more cognitive top-down approach, such as cognitive reappraisal, on sleep physiology after exposure to a stressor. Moreover, the current study focused on the impact of one period of emotion regulation versus repeated experiential emotion regulation and cognitive reappraisal, both relative to the control condition, on negative affect experienced after a stressor and subsequent sleep physiology.

### 4.1. Effects of Experiential Emotion Regulation Versus Cognitive Reappraisal on Negative Affect and Sleep Physiology

In line with daily life experiences of daytime stress and subsequent sleep and consistent with our previous research [10,11], the experimental induction of emotional stress by a failure task worsened the actual experienced mood. Interestingly, the adaptive emotional regulatory impact of the experiential emotion regulation and cognitive reappraisal groups did not differ from each other, nor from the control group, regarding recovery from the pre-sleep negative emotional failure experience. After a first time of emotion regulation, the cognitive reappraisal group did not experience a larger decrease in negative affect, relative to experiential emotion regulation and the control group. However, after a second, repeated emotion regulation moment, experiential emotion regulation did not decrease negative affect more than cognitive reappraisal and the control condition. These findings could possibly be accounted for by the adaptiveness of both emotion regulation strategies and the distracting impact of the reallocation of attention in the control condition as discussed below.

### 4.2. Reallocation of Attention as an Emotion Regulation Strategy

The manipulation check demonstrated the emotional regulatory impact of all three conditions through the decrease in negative emotion after single and repeated experiential emotion regulation and cognitive reappraisal, as well as after the control condition. These results suggest that the reallocation of attention towards the neutral aspects of the task had an emotional regulatory impact. In line with these findings, no significant differences were found in sleep physiology after the repeated deployment of experiential emotion regulation compared to cognitive reappraisal and the control condition.

In the control condition, participants were asked to “Write about the tasks you just completed. Describe them. What were the characteristics of these tasks? You have to focus only on the practical aspects of the tasks.” This process allowed participants to focus their attention on the task itself, its practicalities, and its neutral characteristics. Attention deployment to neutral characteristics, and thus distraction from the emotional experience induced by the task, may have an adaptive emotional regulatory impact [52,53]. In a study by Ferri et al. (2013), the deployment of attention from unpleasant pictures to the less arousing parts of the unpleasant picture resulted in lower activity in brain regions involved with emotions, while prefrontal and parietal activity increased [54]. In addition, by applying this distancing approach, participants may have been more objective in realizing how difficult the task was and, ultimately, may have attributed their poor performance to the task itself, rather than to their intelligence. Moreover, the control condition might also involve some spontaneous or automatic emotion regulation [13,55,56]. Future studies investigating the effectiveness of bottom-up experiential emotion regulation and top-down cognitive reappraisal, and in particular how they differ, should include a more “neutral” control condition that engages less regulation.

### 4.3. Gender Difference in Cognitive Emotion Regulation

Interestingly, non-confirmatory and thus exploratory analyses indicated gender differences in the impact of emotion regulation strategies on follow-up sleep physiology. Interestingly, although there were no gender differences in self-reported negative affect before sleep, sleep physiology of women compared to men benefited more from cognitive reappraisal than from experiential emotion regulation or neutral attention reallocation. Women who deployed cognitive reappraisal showed a longer total sleep time, higher sleep efficiency, a lower percentage of time awake, and fewer awakenings during REM sleep than men. These findings suggest that gender may determine the impact of different emotion regulation styles on subsequent sleep physiology. Moreover, these findings can be interpreted in light of the gender differences in response to negative emotional events in the prefrontal regions, the amygdala, and the ventral striatum [16,57,58,59]. In response to the more intense emotional and physiological reactivity in women [60], the present study showed that women benefit from a more complementary top-down approach such as cognitive reappraisal, relative to men who benefit more from an experiential approach such as experiential emotion regulation. In correspondence, previous research also indicated that applying cognitive reappraisal might be more effortless and potentially more natural for men than women, which could explain why men showed less prefrontal activation [58]. Future research comparing experiential emotion regulation and cognitive reappraisal should further account for these gender differences.

### 4.4. Research Limitations and Future Directions

Despite the number of strengths of this innovative study, the current study contained several limitations too. First of all, as the sample size in the current research was relatively small, including only three between-subject factors (gender, order of nights, and emotion regulation condition), a larger sample size is necessary to provide strong evidence to generalize research findings and increase power. In addition, this dataset contained missing data-points, which further damaged research power and effect size. Secondly, the control condition implemented in the current study was not neutral enough. To this end, we could not meet our aim to determine the effects of each emotion regulation strategy after the failure experience in comparison to a control condition without emotion regulation. Future studies should try to operationalize a more valid control condition whereby the process of induced and automatic dispositional emotion regulation should be limited. Moreover, as we focused on healthy participants who were screened for psychopathological symptoms below clinical levels, the findings cannot be generalized to clinical populations. Future research should assess whether the two emotion regulation strategies would have similar effects in clinical populations, such as those with depression, anxiety disorder, or insomnia. Furthermore, another suggestion for future research is to explore other adaptive strategies to determine a change in the trajectory of emotional responses. By implementing more strategies to compare to, a more differentiated understanding of more adaptive bottom-up and top-down cognitive emotion regulation modes and their related outcomes can be obtained [11,61]. Finally, the effortfulness of polysomnography in the present study to monitor participants’ sleep stages and cycles to identify the moderating effects of emotion regulation strategies, limited the sample size.

## 5. Conclusions

It is well-known that sleep disturbances lead to fatigue, illness, low mental well-being, and psychopathology. As such, research on how to cope with daily life stressors to reduce sleep disturbances by emotion regulation, and how different strategies affect sleep, is critical. The present study explored the adaptiveness of bottom-up experiential emotion regulation versus top-down cognitive reappraisal in reducing negative affect induced by a failure experience, either after single or repeated emotion regulation. Based on the research design and associated results, we can conclude that there is no evidence that the effectiveness of single versus repeated experiential emotion regulation differs from cognitive reappraisal or the control condition by the reallocation of attention towards neutral aspects of the task in the recovery from a failure experience. As the control condition may have induced emotion regulation by attention reallocation and thus distraction, we should further validate the effect of each emotion regulation strategy after the failure experience. Interestingly, the current study suggested that women benefited more from cognitive reappraisal than men, as indicated by less fragmented and arousing sleep.

In conclusion, the present study provides a further step in the experimental exploration of bottom-up experiential emotion regulation as a central working mechanism in experiential-, and client-centered psychotherapy, compared to top-down cognitive reappraisal as a central working mechanism of cognitive psychotherapy. This study also presents novel insights into impacts on the recovery from pre-sleep intense stress and sleep physiology. The findings offer a deeper understanding of the underlying mechanisms in psychological distress and poor sleep quality with substantial implications for treatments and prevention.

## Figures and Tables

**Table 1:** Mean (standard deviation) for age, PSQI and the questionnaire scores under control, experiential emotion regulation, and cognitive reappraisal condition. Mean (standard deviation) for NA and sleep physiology under control, experiential emotion regulation, and cognitive reappraisal condition on the baseline night. Moreover, the obtained F, degree of freedom, and *p* values from one-way ANOVA for each dependent variable were reported ^a,b,c^.

	Control Group (Mean (SD))	Experiential Group (Mean (SD))	Cognitive Reappraisal Group (Mean (SD))	F	Between-Groups df	Within-Groups df	*p* (Two-Tail)
Age	22.27 (3.04)	25 (5.93)	25.77 (5.22)	2.06	2	40	0.14
PSQI	3.00 (1.25)	3.07 (1.39)	3.08 (1.26)	0.02	2	40	0.99
Reappraisal	28.64 (8.70)	27.93 (5.16)	24.23 (6.76)	1.54	2	39 *	0.23
Suppression	12.29 (5.68)	11.60 (3.54)	12.15 (4.10)	0.09	2	39 *	0.91
Emotion processing	21.14 (6.87)	21.33 (6.83)	18.77 (7.11)	0.57	2	39 *	0.57
Emotion expression	21.21 (4.08)	20.53 (3.11)	20.46 (5.52)	0.13	2	39 *	0.88
EACS total	42.36 (9.04)	41.87 (8.00)	40.00 (9.83)	0.26	2	39 *	0.77
DDF	13.57 (2.77)	13.93 (3.73)	13.10 (6.44)	0.11	2	36 *	0.89
DIF	12.79 (3.75)	13.00 (4.12)	11.80 (5.03)	0.26	2	36 *	0.77
EOT	20.18 (6.09)	20.00 (3.70)	20.50 (7.74)	0.02	2	36 *	0.98
Alexithymia	46.50 (8.36)	46.93 (10.57)	45.40 (17.85)	0.05	2	36 *	0.95
NA of baseline night	11.20 (1.52)	11.53 (2.00)	11.92 (2.53)	0.43	2	24.62 #	0.65
Sleep parameters of the baseline night							
% wake	3.88 (2.38)	3.41 (1.99)	5.51 (4.21)	1.25	2	22.57 #	0.31
Sleep efficiency	96.19 (2.39)	96.59 (1.99)	94.49 (4.21)	1.24	2	22.57 #	0.31
Total sleep time	447.0 (15.43)	446.7 (22.12)	430.0 (24.79)	2.77	2	38 *	0.08
Number of awakenings during REM sleep	5.73 (3.90)	5.33 (3.64)	5.69 (5.12)	0.04	2	40	0.96
Number of rapid eye movements	409.8 (222.2)	479.0 (295.5)	529.4 (184.2)	0.664	2	28 *	0.52
Arousal index	5.18 (2.26)	4.94 (2.32)	6.34 (3.51)	0.64	2	12.05 #	0.54
Sleep onset latency	15.03 (14.50)	16.32 (17.78)	24.45 (19.64)	1.13	2	39 *	0.33
Wake after sleep onset	17.73 (11.41)	15.79 (9.03)	25.18 (19.67)	1.15	2	22.42 #	0.34
% N1	4.53 (4.42)	4.87 (4.16)	5.69 (4.31)	0.27	2	40	0.77
% N2	47.48 (10.17)	48.58 (7.41)	49.01 (7.30)	0.12	2	40	0.88
% SWS (N3)	26.49 (7.52)	26.16 (7.89)	20.16 (6.71)	3.14	2	40	0.054
% REM sleep	17.69 (5.05)	15.34 (5.24)	17.48 (3.88)	1.08	2	40	0.35
Latency to REM sleep	104.63 (29.15)	90.63 (44.11)	85.50 (42.81)	0.92	2	40	0.41

^a^ The obtained F, degree of freedom and *p* values from one-way ANOVA for each dependent variable were reported. ^b^ PSQI: The Pittsburgh Sleep Quality Index; EACS: Emotional Approach Coping Scale; DDF: Difficulty Describing Feelings; DIF: Difficulty Identifying Feelings; EOF: Externally Oriented Thinking; NA: Negative Affect. ^c^ * contain missing data points; # Welch test was used instead because Levene’s test for equality of variance was significant.

**Table 2:** Means and standard deviations for emotional experience and objective sleep data for both the baseline and experimental night and for the cognitive reappraisal, experiential emotion regulation, and control condition. Final sample size (N) was also provided for both the baseline and experimental night.

	Baseline Night All Conditions, N	Experimental Night All Conditions, N	Experimental Night Reappraisal, N	Experimental Night Experiential, N	Experimental Night Control, N
**NA of Pre-sleep**					
NA (after baseline movie)	11.53(±2.00), 43	11.84 (±2.96), 43	13.46 (±3.97), 13	11.47 (±2.83), 15	10.80 (±1.01), 15
NA (after failure task)	/	17.02 (±7.31), 43	16.93 (±6.97), 13	17.87 (±8.68), 15	16.15 (±6.63), 15
NA (after ER1)	/	15.19 (±6.58), 43	15.46 (±7.03), 13	15.40 (±7.82), 15	14.73 (±5.09), 15
NA (after ER2)	/	13.86 (±6.12), 43	14.85 (±7.58), 13	13.60 (±7.09), 15	13.27 (±3.41), 15
NA decrease (failure task ER1)	/	1.84 (±2.38), 43	0.69 (±1.97), 13	2.47 (±2.64), 15	2.20 (±2.21), 15
NA decrease (failure task ER2)	/	3.16 (±4.28), 43	1.31 (±3.97), 13	4.27 (±4.48), 15	3.67 (±4.07), 15
**Objective sleep measures**					
% wake	5.58 (±4.12), 42	5.40 (±4.16), 42	6.38 (±5.56), 13	5.00 (±3.40), 15	4.93 (±3.55), 14
Sleep efficiency (%)	95.82 (±2.97), 42	95.57 (±3.44), 42	94.58 (±4.95), 13	96.04 (±2.56), 15	95.91 (±2.68), 14
Total sleep time (min)	441.92 (±21.71), 41	436.22 (±28.76), 41	435.46 (±33.76), 11	439.60 (±22.52), 15	433.58 (±31.97), 15
Sleep onset latency (min)	18.18 (±17.31), 42	19.98 (±14.08), 43	20.39 (±16.55), 13	20.43 (±14.20), 15	19.21 (±12.76), 15
Wake after sleep onset (min)	19.16 (±13.84), 42	22.03 (±20.42), 43	22.97 (±21.54), 13	17.99 (±11.19), 15	25.31 (±26.48), 15
% N1	4.42 (±3.12), 43	4.30 (±3.13), 43	5.50 (±3.42), 13	3.71 (±2.13), 15	3.89 (±3.57), 15
% N2	48.33 (±8.27), 43	48.35 (±9.08), 43	48.67 (±8.59), 13	47.40 (±7.97), 15	48.97 (±10.81), 15
% SWS (N3)	24.46 (±7.79), 43	24.67 (±8.51), 43	21.79 (±8.73), 13	26.54 (±9.60), 15	25.68(±6.92), 15
% REM sleep	16.80 (±4.81), 43	16.42 (±4.37), 43	17.02 (±4.36), 13	15.92 (±4.15), 15	16.41 (±4.81), 15
Latency to REM sleep	93.97 (±39.02),4 3	97.56 (±32.44), 43	95.29 (±37.25), 13	94.53 (±30.74), 15	102.40 (±31.73), 15
Number of awakenings during REM sleep	4.18 (±2.97), 43	4.46 (±3.43), 43	5.42 (±4.95),13	3.96 (±2.56), 15	4.16 (±2.67), 15
Number of rapid eye movements	371.11 (±189.14), 31	350.21 (±194.93), 24	486.13 (±194.50), 5	315.45 (±202.55), 9	336.89 (±132.5), 10
Arousal index	5.28 (±2.78), 39	5.73 (±3.68), 39	6.30 (±4.63), 11	5.82 (±3.66), 14	5.27 (±3.17), 14

**Note** NA = negative affect, ER1 = first time of emotion regulation, ER2 = second time of emotion regulation, NA decrease (failure task ER1) = NA after failure task NA after ER1, NA decrease (failure task ER2) = NA after failure task NA after ER2, % wake = percentage of time awake, REM = rapid eye movement, SWS = slow wave sleep.

## Data Availability

The data presented in this study are available on request from the corresponding author. The data are not publicly available due to the privacy of the participants.

## Supplementary Materials

The following supporting information can be downloaded at: https://www.mdpi.com/article/10.3390/ijerph19137621/s1, Table S1: Correlation matrix of the 15 sleep variables during the experimental night.
