# Supplementary material for: Bottom-Up and Cognitive Top-Down Emotion Regulation: Experiential Emotion Regulation and Cognitive Reappraisal on Stress Relief and Follow-Up Sleep Physiology
Source: Int J Environ Res Public Health. 2022 Jun 22;19(13):7621. doi: 10.3390/ijerph19137621 (PMC9265367; doi:10.3390/ijerph19137621)
Supplement: Supplementary file 1 [file ijerph-19-07621-s001.zip › ijerph-1755766-supplementary.pdf]

Supplementary Table S1. Correlation matrix of the 15 sleep variables during the experimental night[a,b]

Variables	1	2	3	4	5	6	7	8	9	10	11	12	13	14	15
1. Sleep onset latency	1	0.31	-0.77*	0.32	0.11	-0.37	0.35	-0.23	0.18	0.92*	0.75*	-0.22	0.33	-0.35	0.46
2.Wake after sleep onset		1	-0.86*	0.98*	0.42	-0.48*	-0.02	-0.18	-0.04	0.10	0.57*	-0.05	0.74*	-0.98*	0.31
3.Total sleep time			1	-0.81*	-0.39	0.50*	-0.16	0.26	-0.17	-0.57*	-0.80*	0.16	-0.69*	0.83*	-0.45
4.%wake				1	0.18	-0.33	-0.08	0.003	-0.05	0.34	0.40	-0.19	0.84*	-0.995*	0.53*
5.%S1					1	-0.27	-0.15	-0.25	-0.22	-0.06	0.38	0.12	0.23	-0.20	0.14
6.%S2						1	-0.74*	-0.21	-0.08	-0.26	-0.21	0.30	-0.31	0.34	-0.12
7.%SWS(S3)							1	-0.06	0.18	0.33	0.01	-0.26	-0.14	0.08	-0.06
8.%REM								1	0.04	-0.09	-0.42	-0.25	0.05	-0.01	-0.06
9.Latency to S1									1	0.13	-0.01	0.08	-0.01	0.03	0.09
10.Latency to S2										1	0.60*	-0.24	0.27	-0.36	0.47*
11.Latency to SWS											1	0.10	0.36	-0.42	0.37
12.Latency to REM												1	-0.07	0.19	-0.10
13.Number of awakenings during REM sleep													1	-0.84*	0.52*
14.Sleep efficiency														1	-0.52*
15.Arousal index															1

a * indicates $p < 0.003$ with Bonferroni-correction for multiple testing: alpha = 0.003(0.05/15).

b REM = Rapid Eye Movement, SWS = Slow Wave Sleep.
